# Rehmanniae Radix Powder Enhances Antioxidative Capacity, Immunity, and Gut Health in Late-Phase Laying Hens

**DOI:** 10.3390/ani16142122

**Published:** 2026-07-08

**Authors:** Yunqing Guo, Botao Wang, Qingping Luo

**Affiliations:** 1 Key Laboratory of Prevention and Control Agents for Animal Bacteriosis (Ministry of Agriculture and Rural Affairs), Hubei Provincial Key Laboratory of Animal Pathogenic Microbiology, Institute of Animal Sciences and Veterinary Medicine, Hubei Academy of Agricultural Sciences, Wuhan 430064, China; 2 Hubei Hongshan Laboratory, Wuhan 430070, China

**Keywords:** Rehmanniae Radix powder, late-phase, hen, immunity, biochemical indicators, intestinal health

## Abstract

Physiological function is closely linked to production performance in laying hens, especially during the late laying phase. Rehmanniae Radix powder (RRP), derived from the root of *Rehmannia glutinosa* Libosch., exhibits diverse biological activities. We investigated the effects of RRP on immune function in late-phase laying hens. Antioxidant capacity, serum biochemical parameters, splenic immune function, and intestinal health were evaluated. Egg production rate in the RRP group increased by 1.39 percentage points compared with the control group, while average egg weight was not significantly different between groups. Dietary RRP supplementation elevated superoxide dismutase activity and serum albumin level. Splenic expression of immune-related factors, including J-chain, interleukin-2, and caspase-3, was upregulated to varying degrees following RRP supplementation. Helper-to-cytotoxic T-cell ratio also increased, indicating enhanced immune regulation. Intestinal tight junction protein expression was significantly upregulated in the RRP group. RRP also altered relative abundance of intestinal microbes at phylum and genus levels, decreasing *Actinobacteria* abundance and increasing *Bacteroidota* abundance. Similarly, relative abundance of *Bacteroides* and *noank_f_norank_o_Clostridia_UG-014* was elevated. Biological function of the microbial community predictions showed that glycolysis/gluconeogenesis, alanine/aspartate/glutamate, fatty acid biosynthesis, and metabolism were upregulated, while that of the quorum-sensing pathway declined. RRP supplementation improved antioxidant status, splenic immune function, and intestinal health, thereby enhancing physiological conditions in laying hens during the late laying phase.

## Simple Summary

Aging leads to declines in physiological status, including antioxidant capacity, immune function, and intestinal health, in late-phase laying hens, resulting in reduced laying efficiency. In this study, we investigated the effects of dietary Rehmanniae Radix powder on the physiological performance of 540-day-old aged laying hens. Our results revealed that Rehmanniae Radix powder slightly increased the egg production rate and significantly enhanced systemic antioxidant capacity, splenic immune function, and intestinal barrier integrity. While this additive did not alter the overall diversity of gut microbiota, it enriched anti-inflammatory, short-chain fatty acid-generating beneficial bacteria and reduced the abundance of pathobiont microbes. Moreover, it upregulated beneficial pathways such as amino acid metabolism and inhibited the quorum-sensing pathway of pathogenic bacteria. In summary, Rehmanniae Radix powder ameliorates the physiological status of aged laying hens in degrees, providing experimental evidence for its application as a Chinese herbal feed additive.

## 1. Introduction

Eggs are an affordable source of high-quality animal protein and essential nutrients for humans. However, the physiological condition of laying hens declines after approximately 40 weeks of age. This physiological decline in late-laying hens caused by aging mainly involves multiple aspects such as antioxidant capacity, the immune homeostasis of the spleen, and gut health [1,2], and directly suppresses egg production, leading to a decline in substantial economic efficiency [3]. Therefore, improving the physiological condition of hens in order to maintain egg production and extend the laying cycle is critical for improving productivity [4]. Developing interventions to mitigate aging-related physiological decline of aged hens has thus become an urgent industrial demand.

Immune performance, antioxidant capacity, and gut health are the three main physiological modules that affect the overall health of laying hens. First, the spleen acts as the core peripheral immune organ responsible for the proliferation of lymphocytes and immune response regulation. The impairment of splenic function inevitably weakens host resistance against pathogens. Second, antioxidant systems are essential for maintaining overall health by preventing oxidative damage associated with redox imbalances [5]. Oxidative stress has also been recognized as a prominent factor contributing to follicular atresia and ovarian aging [6]. Antioxidant enzyme activity and oxidation product levels are key indicators of oxidative status in poultry. Superoxide dismutase (SOD) plays a crucial role in scavenging free radicals from cells [7]. Third, the intestinal barrier, comprising both physical and microbial components, is essential for protecting the host from pathogens and endotoxins [8,9]. The physical barrier consists of the mucosal layer and intestinal epithelial cells, which form the first line of intestinal defense. The integrity of this barrier depends on tight junction proteins, which are essential structural components [10]. The intestinal microbial barrier comprises the gut microbiota, which contributes to host immune regulation [11]. Thus, splenic immune function, intestinal barrier function, and antioxidant capacity are central to physiological condition.

In light of antibiotic reduction and prohibition in livestock farming, Chinese herbal preparations have emerged as promising feed additives owing to their safety, diverse biological activities and minimal toxic compounds [12]. Rehmanniae Radix (RR), the root of *Rehmannia glutinosa* Libosch. (Scrophulariaceae), is a traditional Chinese medicinal herb with antioxidant, immunomodulatory, and anti-inflammatory properties [13]. It may also influence gut microbiota composition and short-chain fatty acid (SCFA) production [14]. RR has demonstrated considerable therapeutic effects in multiple conditions, including osteoporosis, sepsis-induced acute lung injury, and primary ovarian insufficiency [15,16,17]. It has also been identified as an effective component of herbal formulations that alleviates *Eimeria tenella* infection in chicken [18]. Additionally, polysaccharides from processed RR have been shown to improve tibial ash content by enhancing mineral absorption in broiler chickens [19]. However, the immunomodulatory effects of RR powder (RRP) in laying hens during the production period remain unclear.

In this study, we investigated the effects of RRP on immune function in hens during the late laying stage. The objective was to evaluate the effect of RRP on systemic immunity and related physiological parameters and to provide empirical evidence for its potential to enhance immune function, extend the egg-laying period, and increase economic benefits.

## 2. Materials and Methods

### 2.1. Experimental Design

A total of 324 healthy Dawu Golden Phoenix laying hens (540 days old) were randomly assigned to two groups (control and RRP), each comprising six replicates of 27 hens. The young pullets received a basal diet throughout the rearing period after arrival at this farm, and the composition and nutrient levels of the basal diets are shown in Table A1.

RRP was obtained from Ji Nong Pharmaceutical Group (Shijiazhuang, China). Each gram of processed RRP is equivalent to 2.78 g of raw RR. Following the official product usage guidelines, dietary RRP supplementation was implemented at a dosage of 1 g per hen per day, administered for seven consecutive days (days 1–7) on a monthly cycle, followed by a 23-day withdrawal period. Hens consumed an average of 120 g of feed each day. Accordingly, the RRP group received a basal diet containing 0.83% RRP, whereas the control group was provided with the basal diet only (Table A1). This two-phase cycle was repeated for 2 months. Hens were housed in five-tier ladder-type cages with nine hens per cage, and the replicates were evenly distributed in the cages. The hens were fed twice daily (2:00 and 23:30 h) using an automatic feeding system, and clean water was provided ad libitum. The hens were maintained under a 16-:8 h light/dark cycle at 26 °C with 60–65% humidity.

### 2.2. Egg Production Performance

During the experimental period, feed intake, egg production, and egg weight were measured daily. Egg production rate was calculated as the total number of eggs divided by the number of hens. The feed-to-egg ratio was computed as feed consumption divided by the total egg weight. The average egg weight was calculated as the total egg weight divided by the total number of eggs.

### 2.3. Sample Collection

Blood samples were collected from the pterygoid vein. After incubation at 37 °C for 2 h, serum was obtained by centrifuging the blood at 3000 rpm for 15 min at 4 °C. The cecum, intestinal mucosa, and spleen were collected from hens and stored in cryotubes. The serum, tissue, and cecal content samples were stored at −80 °C until analysis. Simultaneously, portions of fresh spleen and whole blood were used to assay helper (CD4+)/cytotoxic (CD8+) ratios.

### 2.4. Serum Antioxidant Parameters

The total Antioxidant Capacity (T-AOC), Catalase (CAT), and SOD activity were measured using commercial assay kits (Nanjing Jiancheng Institute of Bioengineering, Nanjing, China) according to the manufacturer’s instructions.

### 2.5. CD4+/CD8+ Differentiation

Single-cell suspension of spleen tissue was prepared via mechanical dissociation through a 200-mesh sieve and rinsed with phosphate-buffered saline. The cell suspension was centrifuged at 1000 rpm for 2 min at 4 °C, and then adjusted to 1 × 10^6^ cells/mL. Subsequently, fluorescent monoclonal antibodies CD4-FITC and CD8a-PE were added, and the mixture was incubated in the dark for 30 min. Subsequently, the solution was analyzed using flow cytometry (Beckman Coulter Inc., Brea, CA, USA).

### 2.6. Gene Expression Analysis of Intestinal and Splenic Tissues

Total RNA was extracted from the spleen and intestinal mucosa samples using the FastPure Cell/Tissue Total RNA Isolation Kit (Vazyme Bio, Nanjing, China). Complementary DNA was obtained by reverse transcribing the RNA using the PrimeScript RT Reagent Kit (Takara Bio, Kusatsu, Japan). Quantitative real-time polymerase chain reaction was performed using a Thermo-ABI 7500 system (Thermo Fisher Scientific Inc., Waltham, MA, USA). Reverse transcription was performed using the HiScript II Q RT SuperMix (Vazyme Bio). The primer sequences are listed in Table A2. The 2^−ΔΔCt^ method was used to calculate gene expression. All experiments were performed in triplicate.

### 2.7. Cecal Digesta DNA Extraction and 16S rRNA Sequencing

Cecal contents were collected in sterile tubes and immediately frozen in liquid nitrogen. Total DNA was extracted using a commercial assay kit (Tiangen Biochemical Technology, Beijing, China) according to the manufacturer’s instructions. DNA quality was measured using a Thermo NanoDrop 2000 spectrophotometer (Thermo Fisher Scientific Inc., USA), and DNA integrity was determined using agarose gel electrophoresis (DYY-6C; Beijing Liuyi Biotechnology Co., Ltd., Beijing, China). Qualified DNA samples were used to determine intestinal microbial diversity by Shanghai Majorbic Bio-pharm Technology Co., Ltd. (Shanghai, China).

The process was as follows: The V3-V4 hypervariable region of the bacterial 16S rRNA gene was amplified using primers 338F (5′-ACTCCTACGGGAGGCAGCAG-3′) and 806R (5′-GGACTACHVGGGTWTCTAAT-3′). Purified amplicons were pooled in equimolar amounts and paired-end sequenced on an Illumina Nextseq2000 platform (Illumina, San Diego, CA, USA) according to the standard protocols. Raw sequencing reads were demultiplexed initially, then processed for quality trimming using fastp (v0.19.6; https://github.com/OpenGene/fastp (accessed on 26 September 2022)). Overlapping paired-end reads were assembled with FLASH v1.2.11 (https://github.com/ebiggers/flash/releases (accessed on 21 October 2022)). High-quality sequences were subsequently denoised using the DADA2 plugin in QIIME 2 (version 2024) pipeline with recommended parameters. To eliminate bias caused by uneven sequencing depth when comparing alpha and beta diversity metrics, each sample’s sequence library was rarefied to a uniform threshold of 20,000 reads. Metagenomic function was predicted using PICRUSt2 (Phylogenetic Investigation of Communities by Reconstruction of Unobserved States, v2.2.0).

### 2.8. Statistical Analysis

Data are presented as mean ± standard deviation. Shapiro–Wilk and Levene’s tests were separately performed beforehand to examine whether the data conformed to a normal distribution and possessed homogeneous variances before conducting cross-group comparisons. Differences between groups were analyzed using independent-samples Student’s *t*-test in SPSS software (version 20.0; IBM Inc., Armonk, NY, USA). Differences were considered statistically significant at *p* < 0.05. *: 0.01 < *p* < 0.05; **: 0.001 < *p* < 0.01; ***: *p* < 0.001.

All gut microbiota bioinformatic analyses were carried out via the Majorbio Cloud platform (https://cloud.majorbio.com). Calculations of alpha diversity estimators based on amplicon sequence variant (ASV) profiles were implemented in Mothur v1.30.1 (http://www.mothur.org/wiki/Calculators (accessed on 25 June 2026)). Principal coordinate analysis (PCoA) relying on Bray–Curtis dissimilarity matrices was performed with the *vegan* package in R (v2.5-3) to visualize inter-sample microbial community dissimilarity. Linear discriminant analysis (LDA) effect size (LEfSe) (http://huttenhower.sph.harvard.edu/LEfSe (accessed on 26 April 2026)) was applied to identify the significantly abundant taxa (phylum to genera) of bacteria among the different groups (LDA score > 2, *p* < 0.05).

## 3. Results

### 3.1. Effects of RRP on Egg Production Performance

Egg production rate was significantly higher in the RRP group than in the control group (*p* < 0.05), representing an increase of 1.39 percentage points (Table 1). The average egg weight in the RRP group and control group did not differ significantly (*p* > 0.05). Similarly, no significant difference in the feed-to-egg ratio was observed between the groups (*p* > 0.05). Egg breakage was less than 1% and was therefore excluded from analysis.

### 3.2. Effect of RRP on Antioxidant Capacity

**Figure 1:**
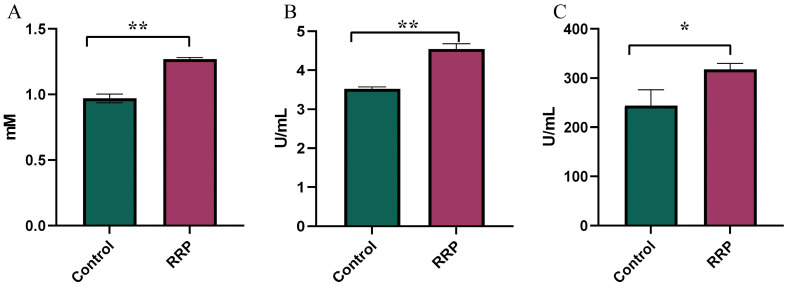
Effects of RRP supplementation on serum biochemical indexes. (**A**) T-AOC; (**B**) CAT; (**C**) SOD. *: *p* < 0.05, **: 0.001 < *p* < 0.01.

Serum T-AOC, CAT, and SOD activity are shown in Figure 1. Serum T-AOC, CAT, and SOD were significantly higher in the RRP group than in the control group (*p* < 0.05).

### 3.3. Effects of RRP on the Immune Index of the Spleen

**Figure 2:**
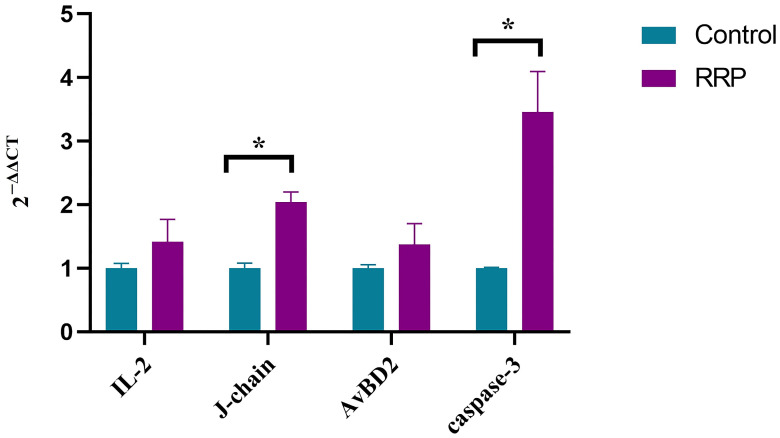
Expression of splenic immune factors. *: *p* < 0.05.

**Figure 3:**
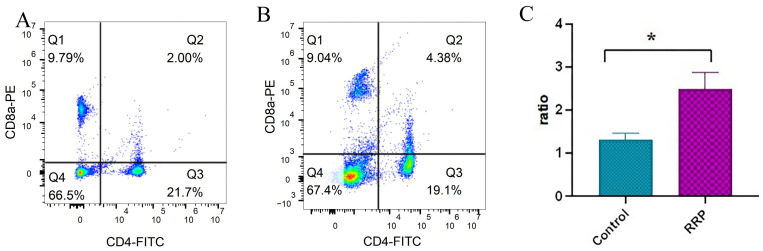
Flow cytometric patterns of CD4+ and CD8+ T lymphocyte expression in the spleen. (**A**) RRP group; (**B**) control group; (**C**) CD4+/CD8+ ratio. *: *p* < 0.05.

The expression of J-chain and caspase-3 was significantly higher in the RRP group than in the control group (*p* < 0.05). By contrast, no significant differences were observed in interleukin-2 (IL-2) and avian beta-defensin 2 (AvBD2) expression between the groups (*p* > 0.05; Figure 2). The CD4+/CD8+ ratio in the RRP group was significantly higher than that in the control group (*p* < 0.05; Figure 3).

### 3.4. Effects of RRP on Gene Expression Related to the Intestinal Mucosa

**Figure 4:**
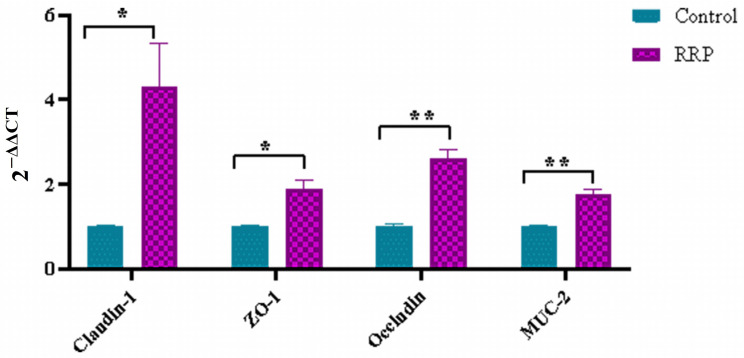
Gene expression related to intestinal mucosa barrier function in the jejunum. *: *p* < 0.05, **: 0.001 < *p* < 0.01.

The expression levels of genes associated with the intestinal mucosal barrier function are shown in Figure 4. In the jejunum, RRP supplementation significantly increased the expression of Claudin-1, Zonula Occludens-1 (ZO-1), occludin, and mucin 2 (MUC-2) (*p* < 0.05).

### 3.5. Effects of RRP on Cecal Microbiota

**Figure 5:**
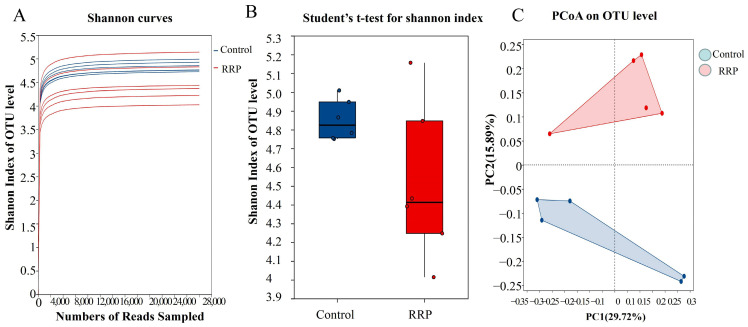
Microbial diversity within groups. (**A**) The Shannon curves; (**B**) alpha-phylogenetic diversity indexes: Shannon index; (**C**) PCoA.

DNA sequences from the 12 fecal samples were analyzed, with a total of 1,107,472 sequences and an average length of 416 base pairs (bps), maintaining an average Good’s coverage of 98.71%. The Shannon curves of the two groups rapidly reached a plateau, indicating that the sequencing data and depth and the sample size were sufficient (Figure 5A). The alpha-phylogenetic diversity based on the Shannon index did not differ significantly between the RRP and control groups (Figure 5B). The beta diversity based on principal coordinates analysis (PCoA) showed that the RRP and control groups were separated (Figure 5C).

**Figure 6:**
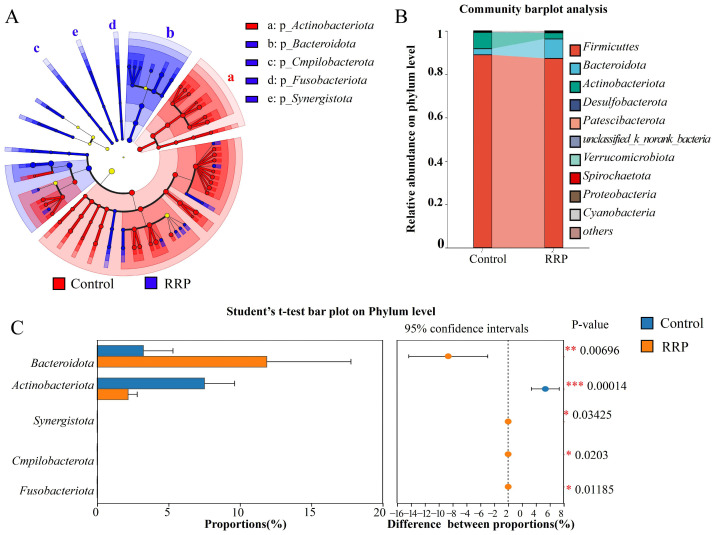
Phylum-level taxonomic composition of the cecal microbiota. (**A**) Cladogram of LEfSe results (from phylum to genus level) for the different groups. The inner to outer circles represent taxonomic classifications from the phylum to genus levels. Phylum labels are indicated on the diagram. (**B**) Phylum-level taxonomic composition of the cecal microbiota. (**C**) The phyla showing significant differences in abundance (*p* < 0.05). *: 0.01 < *p* < 0.05; **: 0.001 < *p* < 0.01; ***: *p* < 0.001.

Linear discriminant analysis effect size (LEfSe) at the phylum level revealed that both groups showed considerable overlap, with significant enrichment within the Firmicutes phylum. While *Actinobacteriota* species were enriched in the control group, *Bacteroidota*, *Synergistota*, *Fusobacteriota*, and *Campilobacterota* species were enriched in the RRP group (Figure 6A).

The predominant phyla of the cecal microbiota in both groups were *Firmicutes*, *Actinobacteriota*, and *Bacteroidota* (Figure 6B). The relative abundance of *Bacteroidota* was significantly higher in the RRP group than in the control group, whereas the relative abundance of *Actinobacteria* was significantly higher in the control group (Figure 6C). Although present at low levels in the cecal microbiome, the relative abundance of *Synergistota*, *Campilobacterota,* and *Fusobacteriota* species also differed between the groups.

**Figure 7:**
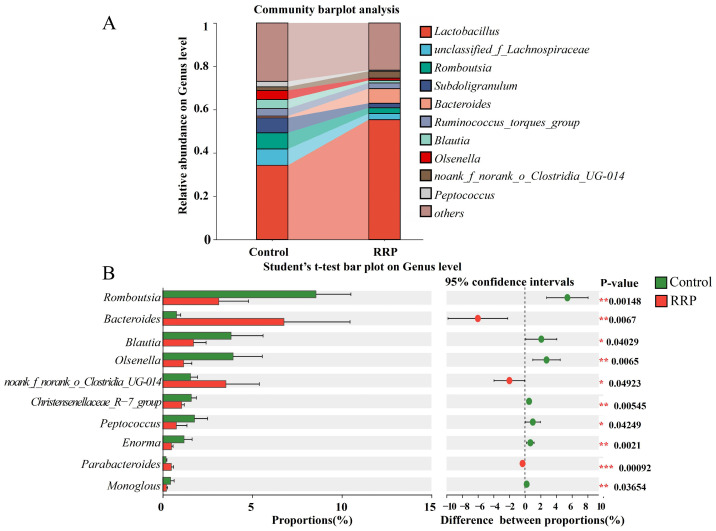
Genus-level taxonomic composition of the cecal microbiota. (**A**) Overall genus-level composition. (**B**) The genera showing significant differences in abundance (*p* < 0.05). *: 0.01 < *p* < 0.05; **: 0.001 < *p* < 0.01; ***: *p* < 0.001.

The overall diversity at the genus level did not differ significantly. The dominant genera of the cecal microbiota in both groups included *Lactobacillus*, *Romboutsia*, *Subdoligranulum*, *Ruminococcus_torques_group*, and *Bacteroides* (Figure 7A). However, significant differences were observed in the relative abundance of several genera (Figure 7B; *p* < 0.05). In the RRP group, *Bacteroides* and *noank_f_norank_o_Clostridia_UG-014* were significantly more abundant, whereas *Romboutsia*, *Blautia* and *Olsenella* were significantly less abundant than in the control group.

**Figure 8:**
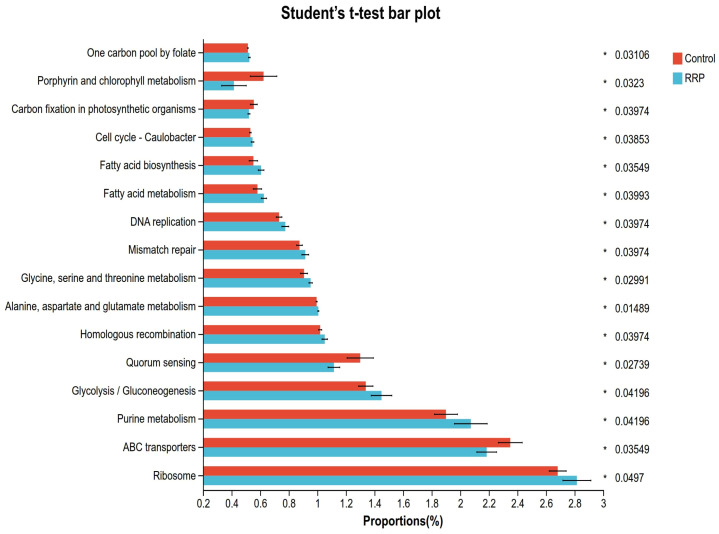
The differences in abundance according to Kyoto Encyclopedia of Genes and Genomes (KEGG) pathway analysis (level 3). *: 0.01 < *p* < 0.05.

For further understanding of the biological function of the microbial community, the metagenomic functions of bacteria were predicted by employing the PICRUSt2 pipeline. The two groups showed significant differences in the Ribosome, ABC transporters, Purine metabolism, Quorum-sensing and some metabolic pathways, including glycolysis/gluconeogenesis, alanine/aspartate/glutamate, fatty acid biosynthesis, and metabolism (Figure 8).

## 4. Discussion

In this study, we investigated the effects of RRP on immune function in laying hens during the late-laying period. RRP supplementation significantly increased T-AOC, CAT and SOD activity. SOD controls the levels of a variety of reactive oxygen species and CAT decomposes hydrogen peroxide, thus limiting oxidative injury, whereas T-AOC represents the integrated antioxidant status of the organism [20,21]. The increased T-AOC, CAT and SOD activity observed in the present study, following RRP supplementation, is indicative of improved antioxidant capacity in laying hens.

As the thymus and bursa of Fabricius regress, the spleen becomes the primary immune organ in late-stage laying hens, contributing to both humoral and cellular immune responses through lymphocytes [22]. In this study, RRP supplementation increased immune factor expression in the spleen to varying degrees. The J-chain is required for the assembly of IgM antibodies [23]. Moreover, the CD4+/CD8+ ratio, an established indicator of immune status, was also higher in the RRP group than in the control group. These findings demonstrate that RRP may enhance splenic immune function during the late laying phase in hens.

The jejunum, the major site of nutrient digestion and absorption in the small intestine, was selected to assess intestinal barrier function. The intestinal barrier includes both physical and functional components critical for maintaining gut health. As the first line of defense, MUC-2 forms a mucus layer that prevents direct contact between luminal bacteria and colonic epithelial cells [24]. Tight junction proteins, including claudin-1, ZO-1, and occludin, are essential for maintaining intestinal integrity [25,26]. In this study, RRP supplementation upregulated the expression of genes encoding these proteins, suggesting improved intestinal barrier function.

The gut microbiota plays a major role in maintaining intestinal barrier function and regulating immune responses [27]. Alpha diversity analysis is primarily an indicator of the richness and overall diversity of microbial communities within a sample, while beta diversity assesses disparities in microbial species composition across distinct samples. These Alpha diversity results indicate that RRP supplementation had minimal effect on the diversity of intestinal microorganisms but influenced the relative abundance of certain bacterial species in laying hens during the late egg-laying stage.

The taxonomic composition of the cecal microbiota on the phylum level shows that *Actinobacteriota*, *Firmicutes*, and *Bacteroidota* were the dominant phyla, which was a consistent pattern observed in laying hens [28]. The LEfSe analysis and the phylum-level relative abundance all showed that *Bacteroidota* were significantly enriched, whereas *Actinobacteria* abundance decreased in the RRP group. *Bacteroidota* can utilize polysaccharides to generate SCFAs. *Bacteroides*, which belongs to *Bacteroidota*, was also enriched in the RRP group. *Bacteroidetes* can increase polysaccharide decomposition to improve nutrient utilization, and regulate inflammatory responses by influencing Treg cell activity [29,30,31]. Moreover, *noank_f_norank_o_Clostridia_UG-014*, belonging to Clostridia, showed increased abundance and the ability to ferment dietary fiber and herbal polyphenols to yield anti-inflammatory SCFA [32]. SCFA reportedly reduces intestinal pH and inhibits the growth of pathogens [33]. This might be a driving factor in RRP enhancing intestinal homeostasis. But the decreased abundance of *Blautia* and *Romboutsia* following RRP supplementation was unexpected. *Blautia* and *Romboutsia* are positively associated with gut health and digestive efficiency [34,35], and their reduction may be compensated by the dramatic expansion of polysaccharide-degrading *Bacteroides*, which undertake overlapping metabolic functions for fiber utilization. In addition, *Actinobacteriota* includes genera such as *Actinomyces odontolyticus*, which can act as pathobionts by secreting genotoxic membrane vesicles that induce DNA damage and drive colorectal cancer development [36]. *Olsenella*, belonging to *Actinobacteriota*, showed decreased abundance in the RRP group.

The enrichment of pathways as predicted by PICRUSt2 provides metabolic-level mechanistic evidence supporting the above taxonomic variations. Primarily, the enrichment of alanine-aspartate-glutamate metabolism, purine metabolism and alanine, aspartate and glutamate metabolism in the RRP group indicated that the RRP group maintained a well-balanced diverse microbiome. The enrichment was caused by the increase in the metabolically active bacteria, such as *Bacteroidota* [37]. Notably, quorum sensing, which regulates virulence traits and biofilm formation in zoopathogenic bacteria, declined in the RRP cecal microbiota [38], which may be a potential pathway for RRP to enhance intestinal homeostasis. However, it should be acknowledged that PICRUSt2 metabolic annotations are computational inferences derived only from 16S rRNA data, and whole metagenomic sequencing is required to validate these differential metabolic pathways.

## 5. Conclusions

In summary, the findings of this study indicate that dietary RRP supplementation enhances the egg production rate, antioxidant capacity, splenic immune capacity, and intestinal mucosal barrier integrity in laying hens during the late egg-laying phase. Although overall diversity was not significantly affected, RRP increased the relative abundance of beneficial genera and was predicted to enhance the metabolic potential of cecal microbes. However, uncovering the specific underlying mechanism requires further investigation.

## Figures and Tables

**Table 1:** Effect of RRP on laying performance of laying hens.

Items	Control Group	RRP Group	*p* Value
Egg production rate (%)	77.59% ± 1.67%	78.98% ± 1.30%	0.02
Average egg weight (g)	63.21 ± 0.93	63.84 ± 1.27	0.19
Feed-to-egg ratio (g:g)	2.37 ± 0.13	2.27 ± 0.09	0.06

## Data Availability

The data presented in this study are available in BioProject at www.ncbi.nlm.nih.gov/bioproject (accessed on 15 May 2026), accession number PRJNA1465579.

## Abbreviations

The following abbreviations are used in this manuscript:

SOD	Superoxide dismutase
T-AOC	total Antioxidant Capacity
CAT	Catalase
PCoA	principal coordinates analysis
LEfSe	Linear discriminant analysis effect size
ZO-1	Zonula Occludens-1
MUC-2	Mucin 2
RR	Rehmanniae Radix
RRP	Rehmanniae Radix powder
SCFA	Short-chain fatty acid

## Appendix A

**Table A1:** Composition and nutrient levels of basal diets.

Item	Content
Ingredients (%)	
Corn	59.5
Soybean meal	19.80
Distillers dried grains with solubles	5.60
Soy sauce grain	3.00
Soybean oil	0.50
Stone aggregates	6.50
Limestone	3.00
Premixal	2.10
Total	100.00
Nutrient levels	
Metabolic energy, MJ/kg	10.88
Crude protein, %	16.5
Methionine, %	0.47
Lysine, %	0.9
Calcium, %	3.80
Phosphorus, %	0.38

**Table A2:** Sequences of the primers used in this study.

Source	Gene Name	Primer Sequence (5′-3′)	Length (bp)
Spleen	IL-2	F:CTTTGGCTGTATTTCGGTAGCA	168
		R:CACTCCTGGGTCTCAGTTGGTG	
	J-chain	F:CATCCTGCTATGCCGAATA	113
		R:TTATACGCTGTCATCCAAGT	
	AvBD2	F:GAGACCAAGAGGTGTTCTG	110
		R:AGTGCCAGGAAGAGGAGA	
	Caspase-3	F:GAGCAGACAGTGGACCAGAT	135
		R:GAGACTGAATAAACCAGGAGC	
Intestinal mucosa	Occludin	F:CGCAGATGTCCAGCGGTTACT	79
		R:CAGAGCAGGATGACGATGAGGAA	
	ZO-1	F:CCACTGCCTACACCATCTC	138
		R:CGTGTCACTGGGGTCCTTCAT	
	Claudin-1	F:GCATGGAGGATGACCAGGTGA	117
		R:GAGCCACTCTGTTGCCATACCAT	
	Muc2	F:CTACTTCACCTTCAACCATTACAACG	163
		R:TCATAGTCACCACCATCTTCTTCTTCAG	
